# A Low Cost Structurally Optimized Design for Diverse Filter Types

**DOI:** 10.1371/journal.pone.0166056

**Published:** 2016-11-10

**Authors:** Majida Kazmi, Arshad Aziz, Pervez Akhtar, Nassar Ikram

**Affiliations:** Department of Electrical Engineering (PNEC), National University of Sciences and Technology (NUST), H-12, Islamabad, Pakistan; Chongqing University, CHINA

## Abstract

A wide range of image processing applications deploys two dimensional (2D)-filters for performing diversified tasks such as image enhancement, edge detection, noise suppression, multi scale decomposition and compression etc. All of these tasks require multiple type of 2D-filters simultaneously to acquire the desired results. The resource hungry conventional approach is not a viable option for implementing these computationally intensive 2D-filters especially in a resource constraint environment. Thus it calls for optimized solutions. Mostly the optimization of these filters are based on exploiting structural properties. A common shortcoming of all previously reported optimized approaches is their restricted applicability only for a specific filter type. These narrow scoped solutions completely disregard the versatility attribute of advanced image processing applications and in turn offset their effectiveness while implementing a complete application. This paper presents an efficient framework which exploits the structural properties of 2D-filters for effectually reducing its computational cost along with an added advantage of versatility for supporting diverse filter types. A composite symmetric filter structure is introduced which exploits the identities of quadrant and circular *T*-symmetries in two distinct filter regions simultaneously. These *T*-symmetries effectually reduce the number of filter coefficients and consequently its multipliers count. The proposed framework at the same time empowers this composite filter structure with additional capabilities of realizing all of its Ψ-symmetry based subtypes and also its special asymmetric filters case. The two-fold optimized framework thus reduces filter computational cost up to **75**% as compared to the conventional approach as well as its versatility attribute not only supports diverse filter types but also offers further cost reduction via resource sharing for sequential implementation of diversified image processing applications especially in a constraint environment.

## Introduction

2D filters are indispensable for many image processing applications such as biomedical [1], industrial [2] and surveillance [3] to name a few. In all of these applications, these 2D-filters (hereafter we call it filters) perform several important tasks such as enhancing fine details, suppressing unwanted noise, detecting edges, image fusion, compression, multi scale decomposition etc. [4–8]. The filtering operation can be performed either in spatial domain or frequency domain however since the images are inherently encoded in spatial domain therefore the spatial domain filtering is preferred [9, 10]. In spatial domain, the filtering operation is carried out by convolving the filter mask with neighborhood pixels of input image [11]. The computation complexity of a (*n* × *n*) filtering operation on a (*M* × *M*) image is *O*(*n*^2^*M*^2^). The quadratic growth of *n* and *M* factors imply that the overall filtering operation is computationally expensive [11]. The implementation of these filters can either be carried out on a software or hardware. Software platforms are slow and time consuming [12] thus not feasible to implement this computationally intensive operation for real time image processing applications. On the other hand, hardware platforms such as Application Specific Integrated Circuits (ASIC) and Field Programmable Gate Arrays (FPGA) are high performance and fast. They satisfy the real time image processing requirements and thus received a great deal of attention for implementing filters in these areas [12, 13].

The conventional approach for implementing a (*n* × *n*) filter on these hardware platforms consume significant computing resources for implementing *n*^2^ multipliers and *n*^2^ − 1 adders [12–14]. This resource hungry solution is not viable especially for the resource constraint environment which are available with limited computing resources, area and power. Therefore the requirement of an efficient and low cost filter implementation calls for exploring different optimization methodologies. Mostly these optimization methods aim to reduce the total multipliers cost because among multiplications and additions, the multiplication operation is more computationally intensive and often becomes a major bottle neck while accelerating the performance of filters on these platforms. There are two main optimization approaches available in open literature for reducing the computational cost of multipliers for these filters. The first approach is based on replacing multipliers with some low cost alternatives, usually by using approximated coefficients value. This coefficient approximation may adversely effects the accuracy of filtered outputs. The second approach is based on reducing the total count of multipliers required by the filter design by reducing effective coefficients count without any change in its value therefore accurately calculates filtered outputs [9, 15–19].

The optimization approaches presented in [9] and [15] were mainly focussed on the cost reduction of multipliers by using low cost arithmetic alternatives. In [9], the hardware cost of multiplier is reduced by using distributed arithmetic approach. This approach replaces traditional multiplication operation with a low cost memory based look-up operation. In this design, no particular property or constancy of coefficients were exploited to optimize operations so it remains fully flexible for implementing filter of any type. The main limitation of this approach is that the coefficients must remain fixed in order to achieve the benefit of cost reduction [20]. Similarly in [15], multipliers cost was optimized by replacing them with a low cost shift-based arithmetic. The drawback of this method is it’s restrict applicability for only those filter types whose coefficients can be expressed in fractional form in such a way that the numerator and denominator of fraction can be expressed in the power of 2. Therefore many useful filters such as Gabor filter and many directional filters cannot be implemented with this approach [15].

Besides the above discussed multiplier less approach, the second approach minimizes the cost of filter by reducing its multipliers count either by exploiting operational or structural properties of the filters [16–19]. An optimized filter design presented in [16] was based on exploiting the operational properties. The 1D separable property of 2D convolution operation was utilized in which filters were separated as one 1D filter in horizontal direction and other 1D filter in vertical direction. It results in reducing the multipliers count for a (*n* × *n*) filter from *n*^2^ multipliers to just *2n* multipliers. The major limitation of this approach is its restrict applicability for only those image processing applications which are comprised of 1D separable filters only whereas a wide range of other applications which are comprised of non-separable filters such as Laplacian, Laplacian of Gaussian, and Difference of Gaussian etc. [10] cannot be implemented with this approach. Furthermore, the second filter optimization technique based on structural properties of the filter was presented in [17] and [18, 19]. The circular symmetry property of filter structure was exploited in [17]. By using this property, the multipliers count for a (*n* × *n*) filter from *n*^2^ multipliers was reduced to $\sum_{k=1}^{i}L_{k}$ where *L*_*k*_ is the *k*^*th*^ layer of multipliers with *k* number of multipliers and *i* = (2, 3, 4, …) for *n* = (3, 5, 7, …) respectively. This design is only applicable for circular symmetric filters. Similarly, the quadrant symmetry property of filter structure was exploited in [19] for an odd sized (*n* × *n*) filter to reduce the multipliers count from *n*^2^ multipliers to $\frac{{\left(n+1\right)}^{2}}{4}$ multipliers. Also in [18], the same quadrant symmetry property was exploited to reduce multipliers count for an even (*n* × *n*) filter from *n*^2^ to one forth i.e. $\frac{{\left(n\right)}^{2}}{4}$ multipliers. The multipliers count for odd sized filters is higher than even sized filters i.e. $\frac{{\left(n+1\right)}^{2}}{4}$. In addition to reduce the multipliers count, they replaced costly multipliers with the low cost logarithmic domain computations for further hardware reduction as compared to the quadrant symmetric design with traditional multipliers. This design is only applicable for quadrant symmetric filters.

The above discussed structurally optimized filter design approaches [17–19] were based on the grouping of similar coefficients within the filter structure. The pixels of input image which are placed at the corresponding symmetric locations of each group are first pre-added and then the resultant pixel value is multiplied with the coefficient of corresponding group. This pre-addition of corresponding pixels of the group before multiplication with coefficient explicitly implies that all the coefficients within that group has same magnitude along with the same sign. Thus all these designs can only be used to implement filters in which the value of coefficients is same with respect to the magnitude as well as with respect to the sign within its symmetry locations, which is actually a subtype of their respective symmetric filters. This way, [17] is capable of implementing only a sub type of circular symmetric filter i.e. identity circular symmetric and so as [18] and [19]implements only identity quadrant symmetric filters [21]. Consequently the scope of all of these structurally optimized designs are narrowed down to implement only a subtype of respective symmetric filters. This common limitation of structurally optimized designs restrict them to incorporate other important anti-symmetric sub-types of these symmetric filters despite of their frequent requirement in many image processing applications and thus make these optimized filter designs infeasible from the application point of view.

It is evident from above literature review that all of the previously proposed filter optimization methods [9, 15–19] strictly constraint their designs for implementing only a small group of specific filters which have those properties that were exploited by the design during optimization. Consequently these optimized designs are compromised to support only those specific image processing applications which utilize filters present within the filter group being supported by them despite of providing an extended support for implementing multiple filter types for fulfilling the demand of advanced image processing applications. On the other hand, the diversified tasks of these image processing applications increasingly require different type of filters simultaneously for processing an input image to achieve their desired results rather than using a single filter type [4–8, 22–27]. These contradictory factors increase the implementation cost of complete image processing application proportionally by using above discussed optimized designs. Therefore to fill this gap, an optimized filter design beyond being getting too specific with respect to filter types is the foremost requirement of all advanced and versatile image processing applications and is the main motivation of this work.

This paper presents an efficient framework for reducing computational cost of filters along with an added aptitude of versatility for implementing a diverse range of filters. The structural properties of filters are exploited to put forward a composite symmetric filter structure in which the identities of quadrant and circular symmetries are applied simultaneously on the two distinct regions of filter structure for effectually reducing its multipliers count. The proposed framework at the same time empowers the composite filter structure with additional capabilities of realizing all of its sub-types and also its special asymmetric filters case thus making it feasible for efficiently implementing diverse filter types for advanced image processing applications. Rest of the paper is organized as follows. Section 2 includes preliminaries of structural symmetry properties of filter. In Section 3 our work is presented. Section 4 includes results and comparison. Section 5 discusses the work. Section 6 includes a comparison of our proposed method with FFT based method whereas section 7 concludes the work. Authors contribution is mentioned in section 8.

## Preliminaries of Structural Symmetry Properties of Filters

**Fig 1:**
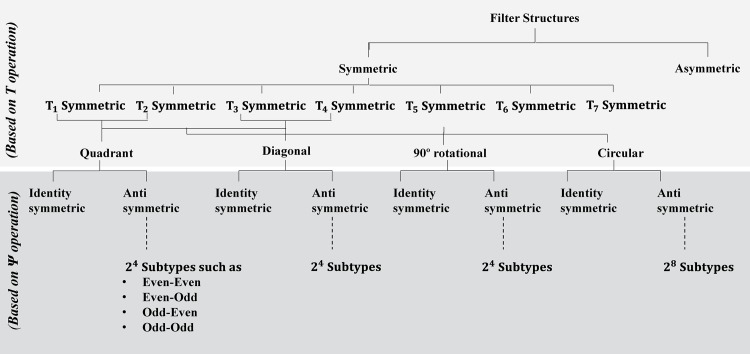
The categorization of filter structure based on *T* and Ψ operations.

A filter structure is *T*-Ψ symmetric if it remain invariant after performing *T* and Ψ operations [21, 28, 29]. These two operations define symmetry within a filter structure *F*(x) over a domain *D* where *T* and Ψ are operations on *x* and on the value of *F*(x) respectively as shown in Eq (1) [21]. The categorization of filter structure based on *T* and Ψ operations is shown in Fig 1.

$$\begin{array}{c} \psi [F(T[x])]=F(x)\;whereas\;x\in D \end{array}$$ (1)

The *T* operation is basically an affine transformation with two fundamental transformations i.e. rotation and reflection. These transformations derive basic *T*-operations on a x_1_,x_2_ plane. Based on these *T*-operations, various *T*-symmetric filter structures are generated as given in Table 1. The number of symmetric regions within these filter structures are determined by the number of cycles of *T*-operation where a k-cyclic *T*-operation is the one that returns original x after k repeated *T*-operation on x i.e. T^*k*^[x] = x.

**Table 1:** Basic *T*-symmetric filter structures.

Transformation	T-operation	Symbol	Symmetric Filter Structure	Mathematical Notation	No of cycle	No of Symmetry regions
Reflection	Reflection about x_1_ axis	T_1_	T_1_-symmetric	F(x) = F(T_1_[x])	2	Two
Reflection	Reflection about x_2_ axis	T_2_	T_2_-symmetric	F(x) = F(T_2_[x])	2	Two
Reflection	Reflection about x_1_ = x_2_ diagonal	T_3_	T_3_-symmetric	F(x) = F(T_3_[x])	2	Two
Reflection	Reflection about x_1_ = -x_2_ diagonal	T_4_	T_4_-symmetric	F(x) = F(T_4_[x])	2	Two
Rotation	90° clockwise rotation about origin	T_5_	T_5_-symmetric	F(x) = F(T_5_[x])	4	Four
Rotation	90° anticlockwise rotation about origin	T_6_	T_6_-symmetric	F(x) = F(T_6_[x])	4	Four
Rotation	180° rotation about origin	T_7_	T_7_-symmetric	F(x) = F(T_7_[x])	2	Two

However in practice, more complex *T*-symmetric filter structures are used in current image processing applications. These complex *T*-symmetric structures are obtained by combining the above mentioned basic symmetric structures. The four most widely used complex *T*-symmetric filter structures in image processing applications are quadrant symmetric, diagonal symmetric, 90° rotational symmetric and octagonal/circular symmetric as given in Table 2 [21, 28]. Among four of them, the first three are 4-cyclic *T*-symmetric structures while the forth one is 8-cyclic *T*-symmetric structure i.e. having four and eight symmetric regions respectively as shown in Fig 1. Therefore coefficient count for a k-cyclic *T*-symmetric filter can be reduced to k-times by considering coefficients of only one region in calculations. Consequently the multiplier count for filtering operation is also reduced by the same factor to multiply reduced number of filter coefficients with pre-added pixels of input image.

**Table 2:** Complex *T*-symmetric filter structures in current image processing applications.

Complex Symmetric filter structures	Conditions for combining basic symmetric filter structures	Number of cycles	Number of Symmetry regions
Quadrant Symmetric	F(x) = F(T_1_[x]) = F(T_1_T_2_[x]) = F(T_2_[x])	Double 2-cyclic	4
Diagonal Symmetric	F(x) = F(T_3_[x]) = F(T_3_T_4_[x]) = F(T_4_[x])	Double 2-cyclic	4
Rotational Symmetric	$\text{F}(\text{x})=\text{F}({\text{T}}_{5}[\text{x}])=\text{F}({\text{T}}_{5}^{2}[\text{x}])=\text{F}({\text{T}}_{5}^{3}[\text{x}])$	4-cyclic	4
Octagonal Symmetric	$\text{F}(\text{x})=\text{F}({\text{T}}_{1}[\text{x}])=\text{F}({\text{T}}_{2}[\text{x}])=\text{F}({\text{T}}_{3}[\text{x}])=\text{F}({\text{T}}_{4}[\text{x}])=\text{F}({\text{T}}_{5}[\text{x}])=\text{F}({\text{T}}_{5}^{2}[\text{x}])=\text{F}({\text{T}}_{5}^{3}[\text{x}])$	Combo of 2 and 4-cyclic	8

These symmetric filter structures are further categorized based on the second operation i.e. Ψ operation. The Ψ operation based symmetries are basically delay type symmetries which alters the sign of the filter coefficients without altering its magnitude [21, 28]. The Ψ operation based commonly known symmetries are identity symmetry and anti-symmetry as shown in Fig 1. If the sign of filter coefficients at all the corresponding locations of symmetric regions is same, then it’s an identity symmetric filter else anti-symmetric filter as given in Eq (2). Based on different coefficients signs at k regions of a k-cyclic filter structure, the anti-symmetric filters are further classified into 2^*k*^ sub-types for example the even-even, odd-odd, odd-even and even-odd are commonly known sub-types of anti-symmetry in image processing for 4-cyclic quadrant filters [29, 30].

$$\begin{array}{ccc} \Psi [F(x)]=F(x) & , & Identity\;symmetry\\ \Psi [F(x)]=-F(x) & , & Anti\;symmetry \end{array}$$ (2)

Conventionally Ψ symmetry is pre-assumed to be an identity symmetry while exploiting *T*-symmetry for reducing the multipliers count in above discussed structurally optimized filter designs [17–19]. The upshot of anti-symmetries in these filter designs is completely disregarded which eventually narrow down their scope to implement only their respective identity symmetric filters.

## Our Work

**Fig 2:**
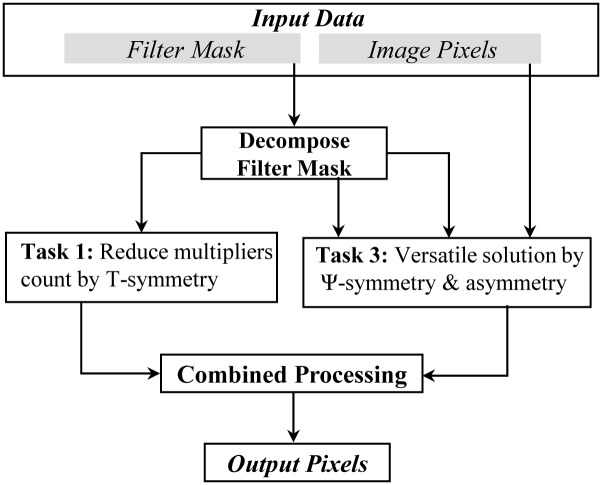
The block diagram of proposed framework for filter design.

This work presents an efficient framework for a low cost and versatile filter design by systematically exploiting *T* and Ψ operations. The block diagram of our proposed framework is shown in Fig 2. In order to exploit these operations, at first the framework decomposes the input filter mask into three distinct sub masks. These sub masks are then processed separately for performing two different tasks i.e. to reduce the multipliers count for a low cost filter design by exploiting *T*-symmetry, and to provide versatility for supporting a diverse range of filter types by incorporating all the sub types of Ψ-symmetry along with asymmetric case. After processing input data separately by *T* and Ψ symmetry operations, their resultants are combined together to provide a low cost and versatile solution.

To perform first task, we introduce a composite *T*-symmetric structure, especially for those filters which are frequently required by many image pre-processing applications such as Gaussian, Laplacian, Laplacian of Gaussian [31, 32], Sobel Compass [33], Sharpening, Smoothening, Frei and Chen [34], etc. The composite *T*-symmetry exploits multiple *T*-symmetries within the multiple regions of filter structure. By combining identities of multiple *T*-symmetries, it significantly reduces the multipliers count for designing a low cost filter. In second task, the Ψ-symmetry based all sub types and an asymmetric case of the composite symmetric filters is also incorporated in the design. The framework considers a special asymmetric case of composite symmetric structure in which if and only if value of its coefficient/s at the corresponding location/s of composite symmetry is zero instead of having the same value of coefficient/s and provides a way to cater this special case within the same filter structure. These two tasks of proposed framework are executed in five steps, which are explained in detail as below.

### The Proposed Framework

**Fig 3:**
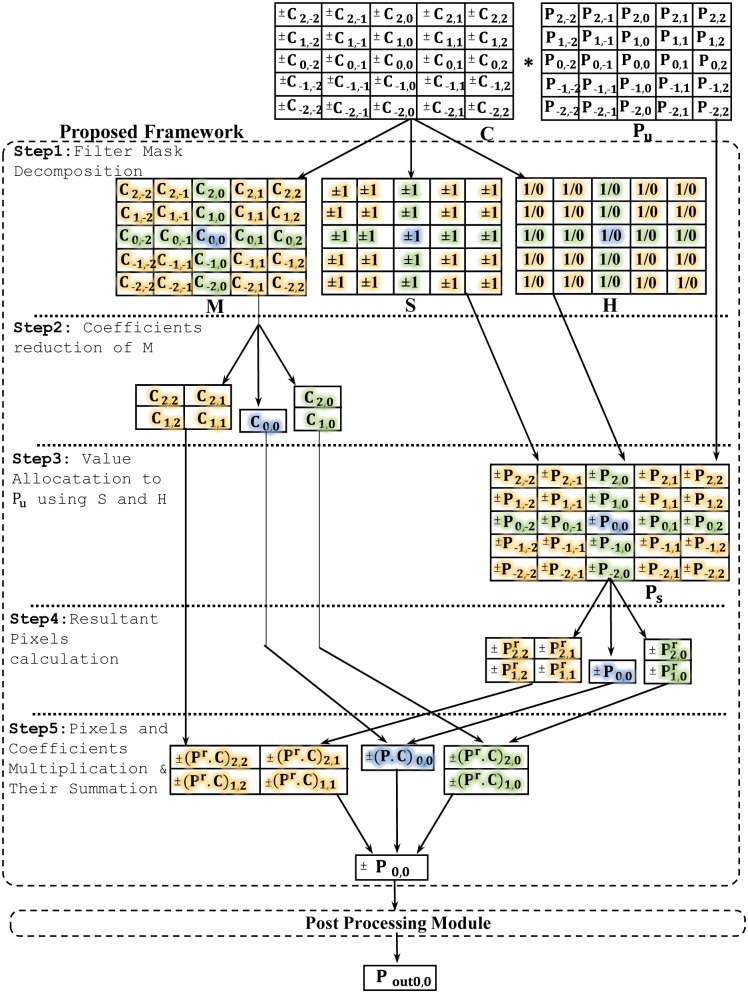
The proposed framework.

Consider a filter mask **C** of size (*n* × *n*); where *n* ≥ 3 and is any natural odd number. The proposed framework performs five steps to convolve **C** with **P**_***u***_ i.e. (a *n* × *n* window of input image pixels) for computing a filtered pixel output **P**_***out***_ as shown in Fig 3. As an exemplary, **C** and **P**_***u***_ are taken as a 5 × 5 window of filter coefficients and input pixels respectively for explanation purpose. The elaboration of each step is given below.

#### Step 1 (Filter Mask Decomposition)

The **C** matrix having coefficients ±C_*x*1,*x*2_ on *x1*, *x2* plane is first decomposed into three distinct matrices **M**, **S** and **H**. The **M** matrix represents magnitude of coefficients, **S** matrix represents signs of coefficients and **H** matrix represent presence of composite symmetry in coefficients as shown in Eqs (3)–(5) respectively. These three matrices **M**, **S** and **H** can be recombined to return **C** by taking their dot product as shown in Eq (6).

$$\begin{array}{c} M=|C| \end{array}$$ (3)

$$\begin{array}{c} S=\{\begin{array}{cc} +1, & \pm \text{C}\geq 0;\\ -1, & \pm \text{C}<0 \end{array} \end{array}$$ (4)

$$\begin{array}{c} H=\{\begin{array}{cc} 1, & \pm \text{C possess proposed composite symmetry};\\ 0, & \pm \text{C}=0\;\text{and does not possess proposed composite symmetry} \end{array} \end{array}$$ (5)

$$\begin{array}{c} \text{C}=\text{M}\cdot \text{S}\cdot \text{H} \end{array}$$ (6)

After decomposing **C** matrix into **M**, **S** and **H** matrices, now these matrices independently exploits different symmetry operations and effectually incorporate their upshots in the design. The magnitude of filter coefficients within **M** matrix exploits identities of multiple *T*-symmetries in multiple regions coded with different colors in Fig 2. The sign of coefficients in **S** matrix incorporates Ψ-symmetry based sub types of *T*-symmetry whereas binary values present at the corresponding location of symmetry in **H** matrix incorporates the special asymmetric case within the design. The distinct symmetric regions in **S** and **H** matrices are also coded with same colors as in **M** matrix.

#### Step 2 (Coefficient Reduction based on *T*-Symmetry by using M matrix)

**Fig 4:**
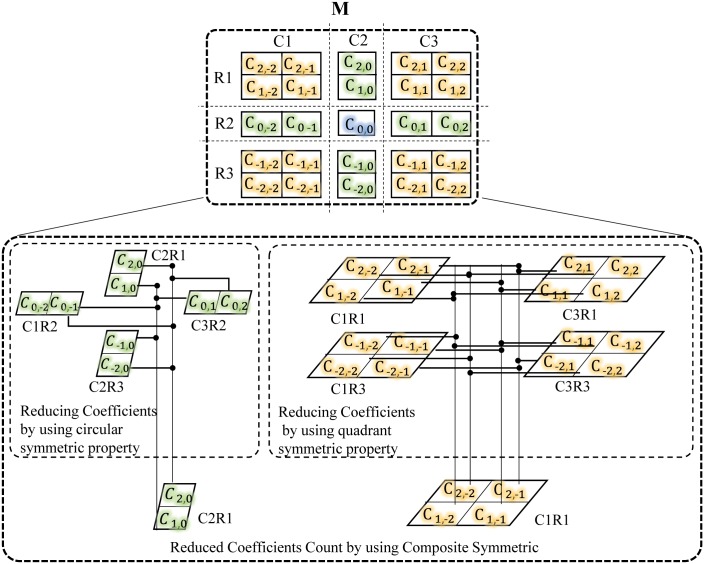
Coefficients reduction within M matrix.

In this step the coefficients count in **M** matrix is reduced down by proposing a composite *T*-symmetry. The proposed composite symmetry is basically the combination of two different *T*-symmetries i.e. quadrant and circular which are found within the two distinct regions of filter simultaneously. These two distinct filter regions are identified by first dividing the **M** matrix in three column wise sub-regions i.e. C1, C2 and C3 and then in three row wise sub-regions i.e. R1, R2 and R3 as shown in Fig 4. The C2R2 is the midpoint having only center coefficient C_0,0_ with no mate as coded in blue color. The rest of the eight sub-regions are grouped into 2 distinct regions. The first region is comprised of C1R1, C1R3, C3R1 and C3R3 sub regions and coded with yellow color while the second region is comprised of C1R2, C2R1, C2R3 and C3R3 sub-regions and coded with green color. The magnitude of those coefficients which are placed in first region are 4-fold quadrant symmetric. Thus for this region, magnitude of coefficients at the corresponding locations of symmetry remains same as defined by the identity of quadrant symmetry in Eq (7) [35].

$$\begin{array}{c} C_{+x1,+x2}=C_{+x1,-x2}=C_{-x1,-x2}=C_{-x1,+x2} \end{array}$$ (7)

Therefore the coefficient of only one sub-region is sufficient to be considered for further computation which ultimately reduce the coefficients count to one fourth in filter design and its computation requirements by the same factor. Now the coefficients of second region are circular symmetric. Therefore for this group, magnitude of coefficients at the corresponding locations of symmetry remains same as defined by the identity of circular symmetry in Eq (8) [35].

$$\begin{array}{c} C_{x1,x2}=constant\;for\;\sqrt{x1^{2}+x2^{2}}=constant \end{array}$$ (8)

Therefore the coefficient of only one sub-region is considered for further computation which reduces the coefficients count to one fourth in filter design and the computation requirements by the same factor. In this way, coefficients spread in the eight sub regions of **M** are reduced to just **25**% of total coefficients as shown in Fig 4 which in turn reduces the number of multipliers by the same factor.

#### Step 3 (Value Allocation to P_*u*_ by using S and H matrices)

This step determines the Ψ-symmetry and the special case of asymmetry in the design by processing **S** and **H** matrices respectively and append it with input pixel matrix **P_*u*_**. The signs of coefficients at the corresponding locations of composite symmetry in **S** matrix determines the Ψ-symmetry of the **C** by using the condition defined in Eq (9).

$$\begin{array}{c} S=\{\begin{array}{cc} \text{if}\;1\;\text{or}\;-1\;\text{on all corresponding locations of symmetry}, & \text{Identity symmetry};\\ \text{if}\;1\;\text{and}\;-1\;\text{on all corresponding locations of symmetry}, & \text{Anti symmetry} \end{array} \end{array}$$ (9)

Based on this condition, the resultant Ψ-symmetry is now incorporated in the design by assigning negative or positive sign of the coefficient to its corresponding unsigned pixels of the **P_*u*_** matrix by taking the dot product of these two matrices. Secondly, in the same step this output is also combined with the **H** matrix. Now the binary value of this **H** matrix represents the presence of special asymmetric case which is incorporated in the design by assigning zero to the corresponding pixels of those coefficient/s which has zero value instead of having same values at the location of composite symmetry in **P_*u*_** matrix by taking the dot product of **P_*u*_** and **H** matrices. The **P_*s*_** is obtained after processing **P_*u*_** with **S** and **H** matrices respectively as shown in Eq (10).

$$\begin{array}{c} {\text{P}}_{s}={\text{P}}_{u}\cdot \text{S}\cdot \text{H} \end{array}$$ (10)

The usage of **P_*s*_** instead of **P_*u*_** in further step empowers the design to incorporate upshot of Ψ –symmetry and the special case of asymmetry within the design.

#### Step 4 (Pre-addition of P_*s*_)

Conventionally, the **P_*u*_** is an unsigned pixels matrix which is used at the pre-adder stage where four unsigned pixels from corresponding locations of symmetry are pre-added and then multiplied with a signed coefficients. The magnitude as well as the sign of coefficients at the corresponding locations of symmetry should remain same for this conventional framework leading to an explicit solution only for identity symmetric filters.

In our proposed framework, unlike conventional approach, now **P_*s*_** matrix is fed to the pre-adders stage. This stage accumulates four signed pixels present at the corresponding location of composite symmetry. The proposed allocation of coefficient signs to pixels at the pre-adder stage results in incorporating upshot of Ψ-symmetry and allows the design to be applicable for all of the sub types of Ψ symmetric composite filters such as even-even, odd-odd, odd-even and even-odd etc. as tabulated in Table 3.

**Table 3:** Resultant pixels computation.

Ψ-symmetry	Resultant Pixel Value $\left(P_{x1,x2}^{r}\right)$
Even-even	(+P_+*x*1,+*x*2_) + (+P_+*x*1,−*x*2_) + (+P_−*x*1,−*x*2_) + (+P_−*x*1,+*x*2_)
Odd-odd	(+P_+*x*1,+*x*2_) + (-P_+*x*1,−*x*2_) + (+P_−*x*1,−*x*2_) + (-P_−*x*1,+*x*2_)
Odd-even	(-P_+*x*1,+*x*2_) + (+P_+*x*1,−*x*2_) + (+P_−*x*1,−*x*2_) + (-P_−*x*1,+*x*2_)
Even-odd	(+P_+*x*1,+*x*2_) + (+P_+*x*1,−*x*2_) + (-P_−*x*1,−*x*2_) + (-P_−*x*1,+*x*2_)

Furthermore as already discussed in step 3, the **H** matrix has assigned zero to the corresponding pixels of those coefficient/s which has zero value instead of having same values on the location of symmetry. Therefore at the pre-adder stage these void pixels nullifies the effect of considering its associated zero valued coefficient as a non-zero symmetric coefficient. This enable the design to also implement the special asymmetric case of composite symmetric filter.

#### Step 5 (Weighted Pixels Computation and Summation)

The reduced set of resultant pixels and coefficient magnitudes at the corresponding location of composite symmetry are now multiplied with each other by using reduced set of multipliers to obtain weighted pixels as shown in Fig 3. The weighted pixels are summed up to get the final pixel value i.e ± P_0,0_. The final pixel is further processed by Post Processing Module in which the absolute final pixel is calculated and then multiplied by scaling factor of filter. Finally, pixel is saturated to input bit level to yield filtered output pixel i.e. P_*out*0,0_.

The proposed framework based on the above discussed five steps leads to a versatile filter solution for implementing all the Ψ based sub-types of composite symmetry and its special asymmetric case. The design has an added advantage of low implementation cost with equal effectiveness on both software and hardware platforms for practical realization. It provide a low cost filter structure for a (n × n) filter by reducing effective coefficient window to just $((\frac{\left(n-1\right)}{2}\times \frac{\left(n+1\right)}{2})+1)$ and consequently the multipliers count to $\frac{\left(n^{2}+3\right)}{4}$.

### Practical Realization of Proposed Framework

**Fig 5:**
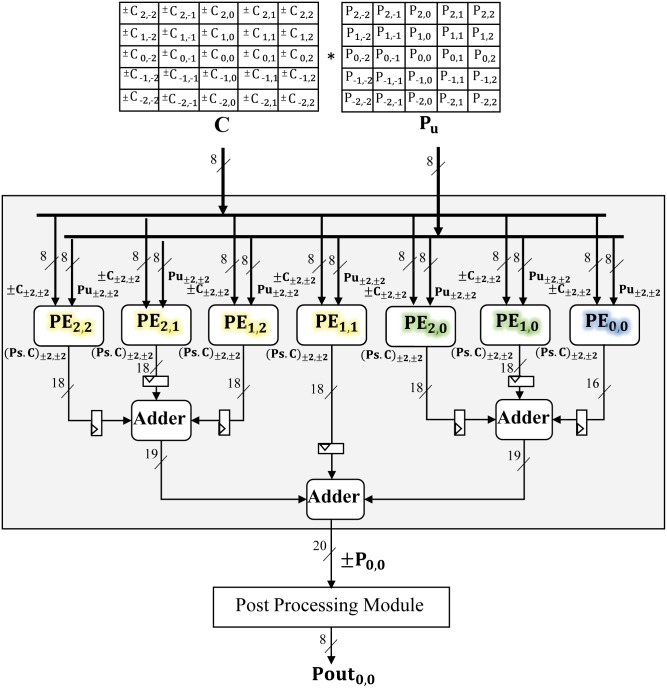
Hardware architecture of an (5 × 5) filter.

In order to practically affirm the effectiveness of proposed filter design framework, a prototype on latest Xilinx FPGA i.e. Artix-7 (XC7A35T) [36] is presented for the above discussed exemplary (5 × 5) filter mask **C**. The hardware architecture of filter is shown in Fig 5. It comprises of a set of seven (i.e. $(\frac{\left(5^{2}+3\right)}{4}=7$) parallel pipelined Processing Elements (PE_*x*1,*x*2_)and an adder tree. The design acquires coefficient and pixel array i.e. **C** and **P_*u*_** as input and give filtered pixel P_0,0_ as output.

Two input data arrays, an array of 8 bit coefficients i.e. ±C_±*x*1,±*x*2_[7:0] of **C** and the second array of 8 bit grey scale input pixels i.e. Pu_±*x*1,±*x*2_[7:0] of **P_*u*_** is acquired from on-chip memory and fed to these seven PEs_*x*1,*x*2_ i.e. PE_0,0_, PE_1,0_, PE_1,1_, PE_2,0_, PE_1,2_, PE_2,1_, and PE_2,2_. These PEs are the basic building block of our hardware design and are purposely designed for performing all the five steps of proposed framework on input data except the final summation.

The PE_2,2_, PE_1,1_, PE_2,1_, and PE_1,2_ (marked as yellow) process four sets of quadrant symmetric coefficients i.e. ±C_±2,±2_, ±C_±1,±1_, ±C_±2,±1_, and ±C_±1,±2_ of input **C** with their corresponding input pixels i.e. Pu_±2,±2_, Pu_±1,±1_, Pu_±2,±1_, and Pu_±1,±2_ of input **P_*u*_** respectively to compute weighted pixels i.e. ±(P.C)_2,2_, ±(P.C)_1,1_, ±(P.C)_2,1_, and ±(P.C)_1,2_. Similarly, the PE_1,0_ and PE_2,0_ (marked as green) process two sets of circular symmetric coefficients i.e. ±C_±1,0_, ±C_±2,0_ with Pu_±1,0_, Pu_±2,0_ to compute ± (P.C)_1,0_ and ± (P.C)_2,0_ respectively. The PE_0,0_ (marked as blue) process the center coefficient of C_0,0_ with Pu_0,0_ to compute ± (P.C)_0,0_. The seven intermediate outputs of these seven PEs are summed up by using three 3-input adders arranged in a tree to get the final output i.e. ± P_0,0_.

**Fig 6:**
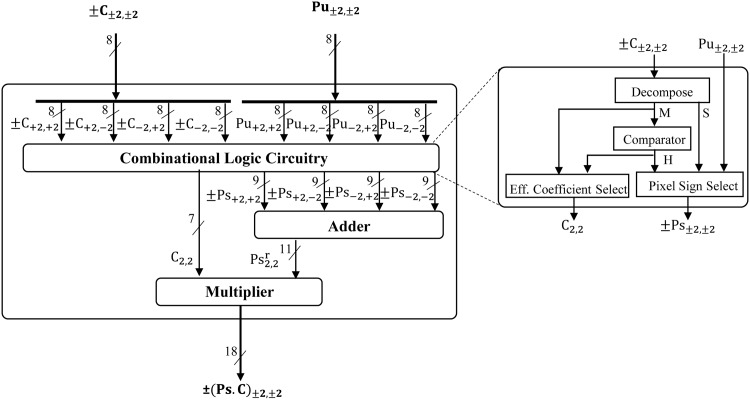
Internal architecture of processing element (PE_2,2_).

The six out of total seven PEs_*x*1,*x*2_ (i.e. PE_1,0_, PE_1,1_, PE_2,0_, PE_1,2_, PE_2,1_, and PE_2,2_) are identical and has similar internal architecture. Therefore the detail diagram of only one PE i.e. (PE_2,2_) and its corresponding set of data is elaborated in Fig 6. It consists of three main units i.e. a combinational logic circuitry, a 9 bit 4-input adder and a (7x11) multiplier. For processing input data it registers the four quadrant symmetric coefficients i.e. ±C_+2,+2_, ±C_+2,−2_, ±C_−2,+2_, ±C_−2,−2_ of **C** and its corresponding input pixels i.e. Pu_+2,+2_, Pu_+2,−2_, Pu_−2,+2_ and Pu_−2,−2_ of **P_*u*_** and fed to its combinational logic circuitry.

The first three steps of proposed framework (i.e. Filter mask decomposition, coefficient reduction of **M** and value allocation to **P_*u*_**) are performed by this combinational logic circuitry as shown in Fig 6. At first it decomposes the four quadrant symmetric coefficient inputs into its four corresponding values in **M**, **S** and **H** arrays (matrices) by using the selection logic based decomposition module. Its selection logic is tabulated in Table 4. For finding the **M**, absolute value of input signed coefficients is computed by making the selection between input and its 2’s compliment where Most Significant Bit (MSB) of input coefficient is served as the selection pin. The same selection module also selects positive or negative sign for the corresponding locations in the **S** array (matrix). These four acquired absolute values of symmetric coefficients are now compared with each other by using a comparator, to compute the corresponding binary values of the **H** array (matrix). It compares the absolute coefficient on a specific location with the rest of other three symmetric absolute coefficients. If it is equal to the three compared coefficients or if it is a non-zero number then its corresponding value in the **H** is 1 else it is 0.

**Table 4:** Selection logic for decomposing C into M and S.

Input ±C_±2,±2_	Select (MSB of input)	Output M_±2,±2_	Output S_±2,±2_
±C_+2,+2_	0	±C_+2,+2_	1
	1	2’s complement ±C_+2,+2_	−1
±C_+2,−2_	0	±C_+2,−2_	1
	1	2’s complement ±C_+2,−2_	−1
±C_−2,+2_	0	±C_−2,+2_	1
	1	2’s complement ±C_−2,+2_	−1
±C_−2,−2_	0	±C_−2,−2_	1
	1	2’s complement ±C_−2,−2_	−1

Now after computing four corresponding values in **M**, **S** and **H** arrays (matrices), the combinational logic assigns appropriate signs (in case of symmetric)or a zero (in case of asymmetric) to the four input unsigned pixels i.e. Pu_+2,+2_, Pu_+2,−2_, Pu_−2,+2_ and Pu_−2,−2_; by using pixel sign selection module. It assigns a value to the output Ps which will be either the input Pu, 2’s compliment of Pu or a zero by using **S** as **H** as shown in Table 5. Finally a single symmetric absolute coefficient is selected among the four input coefficients provided that it has a unity value for its corresponding **H**. This single coefficient is now transmitted to perform calculations in further steps of framework. Therefore reducing the effective coefficient from 4 to just 1 and the further hardware requirements by the same factor for processing further steps.

**Table 5:** Pixel sign selection logic for computing Ps_±2,±2_.

Input Pu_±2,±2_	Select		Output Ps_±2,±2_
	S_±2,±2_	H_±2,±2_	
Pu_+2,+2_	x	0	0
	1	1	Pu_+2,+2_
	−1	1	2’s complement Pu_+2,+2_
Pu_+2,−2_	x	0	0
	1	1	Pu_+2,−2_
	−1	1	2’s complement Pu_+2,−2_
Pu_−2,+2_	x	0	0
	1	1	Pu_−2,+2_
	−1	1	2’s complement Pu_−2,+2_
Pu_−2,−2_	x	0	0
	1	1	Pu_−2,−2_
	−1	1	2’s complement Pu_−2,−2_

Now the above calculated four 9 bit signed pixels Ps_+2,+2_, Ps_+2,−2_, Ps_−2,+2_ and Ps_−2,−2_ from the combinational logic circuitry are summed up by using a 9-bit 4-input adder (as in step 4 of framework). Finally this resultant 11 bit signed output pixel is multiplied with the above calculated 7 bit unsigned coefficient to compute the weighted pixel ± (P.C)_2,2_ by using a (7x11) multiplier(as in step 5 of framework). This weighted pixel is taken as an output of the PE and transmitted to the adder tree for final summation.

Unlike to the above mentioned description for six identical PEs, the seventh PE i.e. PE_0,0_ process the center coefficient with center input pixel which has no symmetrical mate. Therefore its processing is simplified on hardware by bypassing all five steps and directly multiplying input ±C_0,0_ with P_0,0_ to compute the weighted pixel ± (P.C)_0,0_ by using a single multiplier. The output of these seven PEs are then summed up by using 18 bit 3-input adders to get filtered pixel value as shown in Fig 5.

Our complete filter design requires seven multipliers and ten adders (multi input adders are used to get the benefit of compressor tree logic on FPGA [37]) along with a low cost combinational logic circuitry. Since the complete combinational logic circuitry is based on selection and comparison logic and thus mainly comprised of multiplexers and comparators units. These units are light weight hardware components and thus consume a small amount of logic Slices on FPGA hardware. Therefore the complete design occupies only 489 logic Slices on target FPGA device with an operating frequency of 314 MHz. The achieved operating frequency is high enough to sustain high frame rates for real-time image processing.

## Results and Comparison

In this work we present a structurally optimized filter design along with an additional aptitude of versatility. The structural optimization reduces the multipliers count required for performing filtering operation. Unlike the conventional filter structural optimization approaches [17–19] which though reduces the multipliers count but consequently narrow down the scope of design for implementing limited filter types, the proposed framework reduces the multipliers count and at the same time is capable of incorporating diverse range of filter types. It increases its effectiveness for implementing diversified image processing applications.

To quantify our results let us consider a (*n* × *n*) filter mask which performs filtering operation on a (*M* × *M*) image. The computaion cost in terms of total multipliers count for our proposed design and its comparison with other structural optimized approaches is shown in Table 6. The conventional un optimized filter structure requires *n*^2^ multipliers for multiplying (*n* × *n*) filter coefficients window with (*n* × *n*) input image pixels per output pixel calculation [14]. The quadrant symmetry based optimization [18, 19] reduced the multipliers count from *n*^2^ to $\frac{\left(n^{2}+2n+1\right)}{4}$ by reducing effective coefficients window from (*n* × *n*) to $\frac{\left(n+1\right)}{2}\times \frac{\left(n+1\right)}{2}$. While our proposed composite symmetric structure further reduces the multiplier count to $\frac{\left(n^{2}+3\right)}{4}$ by further reducing coefficients window to $((\frac{\left(n-1\right)}{2}\times \frac{\left(n+1\right)}{2})+1)$ by the virtue of exploiting multiple *T*-symmetries simultaneously. By reducing multipliers count, the computational complexity in terms of total multiplication operations required to process an input image is also reduced by the same factor as tabulated in Table 6.

**Table 6:** Results and comparison.

Method	Multipliers Count	Computational Complexity	Total MOPS for a real time application
*Conventional*	n^2^	O(n^2^ × *M*^2^)	3858.4 MOPS
*Quadrant*	$\frac{n^{2}+2n+1}{4}$	$\text{O}(\frac{n^{2}+2n+1}{4}\times M^{2})$	1425 MOPS
*Proposed*	$\frac{n^{2}+3}{4}$	$\text{O}(\frac{n^{2}+3}{4}\times M^{2})$	1108.3 MOPS

The implication of this cost reduction is substantial for realizing practical applications. In order to affirm it, let us apply the above acquired results for practical realization of a (5 × 5) filter on a full HD 1080 × 1920 input image for a real time image processing application. With an operating frequency of 314 MHz, our filter design will sustain the frame rate of 151 fps. At this frame rate, the conventional filter structure requires **3858.4** Million multiplication Operations Per Second (MOPS) and quadrant filters requires **1425** MOPS while our proposed composite symmetric filter require only **1108.3** MOPS as shown in Table 6. This reduction in MOPS is due to the fact that our proposed framework requires least number of multiplication operations which will ultimately reduces the computational burden and power as compared to the other two approaches. It is evident from these results that our proposed filter design is capable of reducing the multipliers requirement of filters up to **75**% and **25**% as compared to the conventional and quadrant symmetric filters respectively.

Since this significant performance gain is obtained only by reducing the multiplications associated with the redundant symmetric coefficients without approximating the value of coefficients therefore the proposed method has not induces any error in calculating the output. Thus no artifacts in the resultant filtered image.

## Discussion

**Fig 7:**
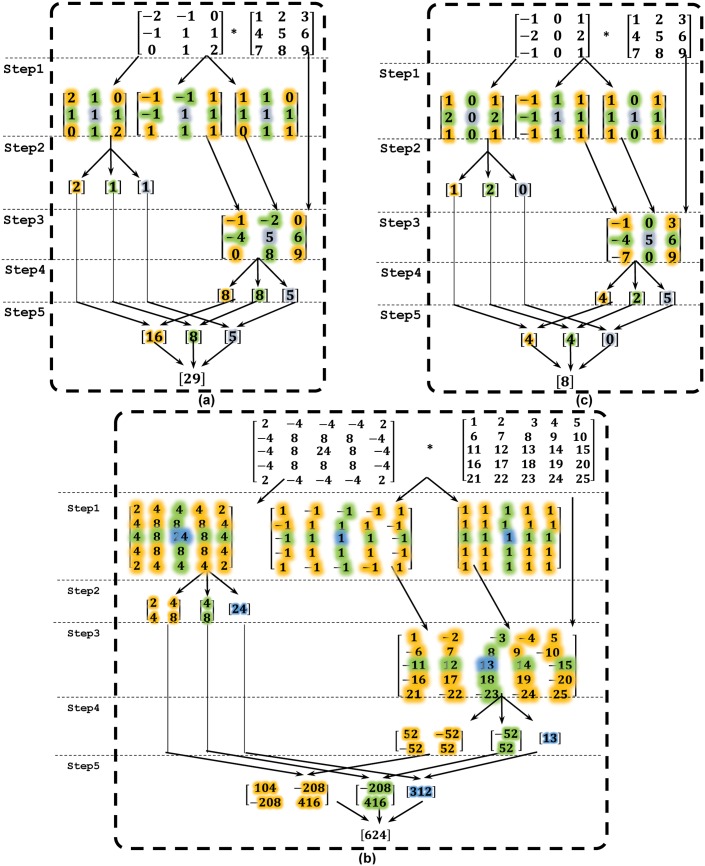
Realization of distinct filter types by using proposed framework. (a) 3x3 Emboss filter. (b) 5x5 Laplacian of Gaussian filter (c) 3x3 Sobel-X filter.

It is evident from results comparison that the proposed composite symmetric structure significantly reduced multipliers count to yield a compact filter design. However, beside reducing multipliers count, the major advantage of our proposed framework is its aptitude of realizing a diverse range of filter types due to incorporating Ψ-symmetry and the special asymmetric case of composite filter structures. While on the other hand the previously reported filter optimization approaches [18, 19] though minimize the multipliers count but at the same time limits its scope for realizing a specific filter type. The effective realization of diverse filter types by using our framework is elaborated in Fig 7(a)–7(c) in which three unlike filter types are chosen for the realization. In Fig 7a, a 3 × 3 Emboss filter is realized which is a diagonal anti-symmetric filter, while in Fig 7b a 5 × 5 Laplacian of Gaussian is realized which is an identity circular symmetric filter. Similarly in Fig 7c, a 3 × 3 Sobel-X filter is realized which is an anti-quadrant symmetric filter. By virtue of versatility, the proposed framework is capable to realize all of these entirely different filter types as composite symmetric filter.

In Fig 7a, a 3 × 3 Emboss filter mask **C** is convolved with a 3 × 3 input pixels window **P_*u*_** to yield a filtered output pixel. It systematically performs all the five steps of proposed framework as discussed in section 3. Step 1 decomposes Emboss filter mask into three matrices i.e. **M**, **S** and **H** matrices. Step 2 reduces filter coefficients of **M** matrix by using composite symmetry from **9** coefficients to just **3** coefficients. Now the unlike signs of unit values in **S** matrix at the corresponding symmetry locations depicts that Emboss is an anti-composite symmetric filter (refer to Eq (9)). Furthermore two 0’s in 3 × 3 **H** matrix at the third column of first row and first column of third row represents that it is the special asymmetric case of composite symmetric filter. Step 3 incorporates the upshot of Ψ-symmetry and asymmetry in the design by appending **S** and **H** matrices within unsigned pixels matrix **P_*u*_** before their pre-addition. It assigns sign of coefficients to its corresponding unsigned pixels and also assigns void pixels at third column of first row and first column of third row. Step 4 is a pre-adder stage where processed pixels of **P_*s*_** matrix at the corresponding symmetry locations are added to yield **3** resultant pixels. The resultant pixels has incorporated Ψ-symmetry and asymmetry in design. The step 5 involves multiplication of **3** coefficients with **3** resultant pixels and their summation. Its output is fed to Post Processing Module for further processing.

In Fig 7b 5 × 5 Laplacian of Gaussian filter mask **C** is convolved with a 5 × 5 input pixels window **P_*u*_**. Step 1 decomposes the filter mask **C** into **M**, **S** and **H** matrices. Step 2 reduces filter coefficients of **M** matrix from **25** coefficients to just **7** coefficients. For this identical composite symmetric filter, the sign of unit values in **S** matrix at all the corresponding symmetry locations are same and its **H** matrix is a 5 × 5 identity matrix. The rest of the steps are performed in same way as for Fig 7a. Similarly in Fig 7c a 3 × 3 Sobel-X filter mask **C** is convolved with a 3 × 3 input pixels window **P_*u*_**. Step 2 reduces filter coefficients from **9** coefficients to **3** coefficients. This anti-composite symmetric filter has unlike signs of unit values in **S** matrix at the corresponding symmetry locations and 0’s in its **H** matrix. The rest of the steps are performed in the same way as for Fig 7a.

**Fig 8:**
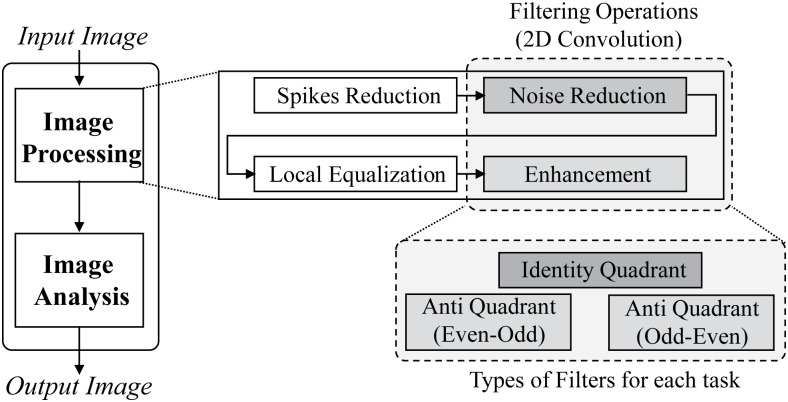
Data flow diagram of chosen biomedical image processing application.

The realization of above exemplary filters of diverse types clearly shows the versatility of our proposed framework along with reducing the multipliers count by the same factor for all of them. This versatility attribute itself has two-fold advantages. Firstly it provides the capability within a single design for efficiently implementing a wide range of filter types and secondly it offers a further cost reduction via resource sharing for implementing those image-processing applications which require multiple types of filters sequentially for performing diversified image processing tasks. The diversified filter requirement for different applications ranges from biomedical [4, 5, 22, 23], computer vision [6, 24], surveillance and navigation [7, 25], industrial [26, 27] to geophysics [8] etc. In contrast to our proposed versatile framework, the previously reported structurally optimized designs such as [18] and [19] does not offer further cost reduction for such applications. These designs [18, 19] though reduces the multiplier cost but are incapable to incorporate their respective anti-symmetric filters. Therefore in order to apply theses designs for anti-symmetric filters, some permanent modifications are mandatory in their existent design on type to type basis which leads to the requirement of as many distinct filter designs for as many distinct types. These requirements restrict them from resource sharing and thus eliminate the possibility of further cost reduction. In order to affirm the effectiveness of versatility of proposed design in terms of the cost reduction for these applications, let us consider an application from the biomedical image processing area in which different types of filters are required for processing an input ultrasound image [22]. Their design is comprised of two different type of filtering tasks, one for the noise removal and other for the image enhancement. To accomplish these two diversified filtering tasks it requires one 3 × 3 image smoothening filter, and two 3 × 3 Sobel filters i.e. one in horizontal and other in vertical direction respectively. All of these three filters are of different types, the smoothening filter is a circular identity-symmetric filter (A subset of quadrant-identity filters [18]) while the two Sobel filters are quadrant anti-symmetric (one is Even-Odd and the other is Odd-Even) filters. The data flow diagram of the complete application algorithm [22] along with its filtering requirement is shown in Fig 8

For practical realization, all of these three filtering operation can be executed by using parallel/pipelined architecture, sequential architectures or the combination of these two, depending on the computational time and area requirements (constraints) of the design. For a parallel architecture, all the filtering tasks are performed concurrently by using a separate filter for each task with pipelined stages. This decreases the overall computation time for the complete design at the expense of increasing overall area requirements. While for a sequential architecture all of these filtering tasks need to be executed sequentially in a time multiplexed way preferably by using a single filter. This in turns reduces the overall area requirements at the expense of increasing computational time for the complete design. Alternatively these filtering operations can be executed by using the combination of parallel and sequential architecture. For all these architectures the total area requirement in terms of multipliers count can be calculated by using Eq (11).

$$\begin{array}{ccc} Total\;cost & = & \sum_{i=1}^{p}\frac{TMS}{s} \end{array}$$ (11)

*where*

$$\begin{array}{ccc} p & = & Parallel\;filter\;connections\;whereas\;1\leq p\leq N\\ s & = & Sequential\;filters\;whereas\;1\leq s\leq N\\ TMS & = & Total\;Multipliers\;of\;Sequentially\;connected\;filters\\ N & = & Total\;filters\;utilized \end{array}$$ (12)

**Fig 9:**
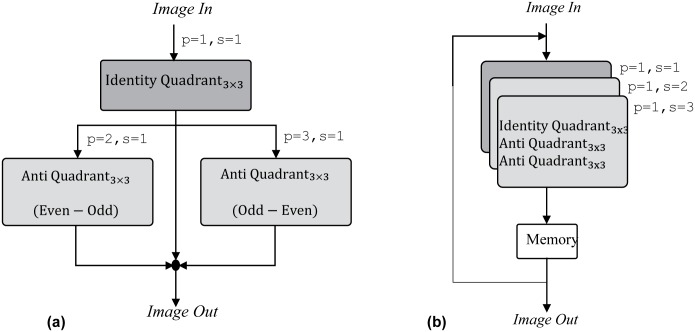
An example of biomedical image processing application. (a) Parallel architecture. (b) Sequential architecture.

**Fig 10:**
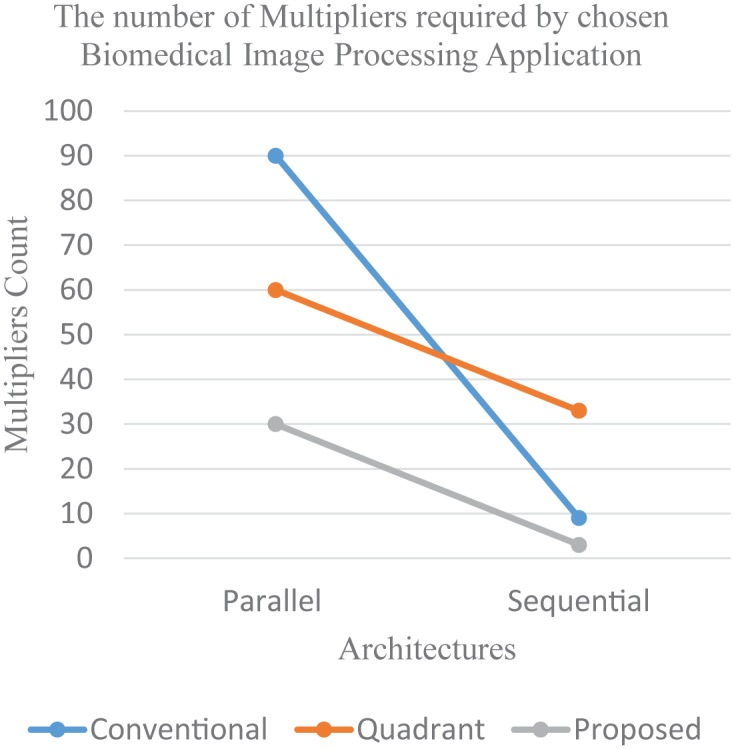
Total multipliers for parallel and sequential architectures of chosen biomedical application.

Now let us consider the parallel/pipelined architecture for the implementation of chosen biomedical application by using conventional, quadrant and our proposed filter design. Three separate filters of three different types (identity quadrant, anti-quadrant (Even-Odd) and anti-quadrant (Odd-Even) are required for performing the filtering tasks concurrently as shown in Fig 9a. The total multiplier cost for the complete design is calculated as $\sum_{1}^{3}9/1=27$, $\sum_{1}^{3}4/1=12$ and $\sum_{1}^{3}3/1=9$ multipliers for conventional, quadrant symmetric (after applying modifications for anti-quadrant filters at the pre-adder stage) and our proposed filter design approaches respectively. Now for implementing the same application sequentially, it iteratively requires a single filter as shown in Fig 9b. The design of this single filter must be capable to implement all required types of filters. This aptitude if offered by versatile filter design approaches such as conventional filter designs and our proposed filter design while the fixed quadrant symmetric filter design is unable to fulfill this condition therefore is not a feasible option for sequential architectures despite of the fact that these architectures are specifically chosen for implementing applications in area constraint environment and for which optimizations in filter designs are meant to be made. The total multiplier cost for the complete design with conventional and our proposed approach is calculated as $\sum_{1}^{1}27/3=9$ and $\sum_{1}^{1}9/3=3$ multipliers respectively. However for the sequential architecture of quadrant symmetric filter design, its multiplier count will remain same as for its parallel architecture $\sum_{1}^{3}4/1=12$. The total multipliers count of parallel and sequential architectures of chosen application is plotted in Fig 10 which affirms that versatility of proposed filter design approach has a big impact on cost reduction for sequential architectures of applications. The optimized yet fixed quadrant optimized filter design approach behaves worse than conventional un-optimized approach in this case due to inability of getting benefit by resource sharing of multiple filter types.

**Fig 11:**
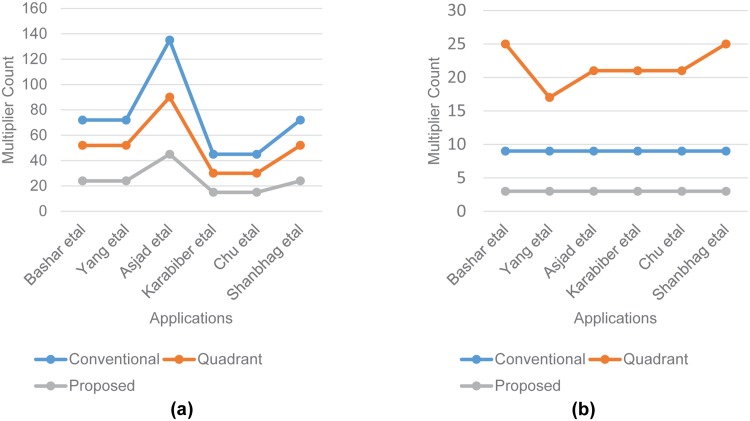
Total multipliers count for different image processing applications. (a) Parallel architecture. (b) Sequential architecture.

Similarly, the trend of cost reduction will almost remains same for other image processing applications [4–8, 23–27] either implemented as parallel or sequential architecture. The [6] and [23] applications are comprised of four anti-quadrant symmetric filters and four non-quadrant symmetric filters for performing respective filtering tasks. The [27] requires two anti-quadrant symmetric filters and two non-quadrant symmetric filters. The [8] requires three identity quadrant symmetric filter, six anti-quadrant symmetric filters and six non-quadrant symmetric filters. The [25] and [24] require an identity quadrant symmetric filter, two anti-quadrant symmetric filters and two non-quadrant symmetric filters. Fig 11a shows that for implementing parallel filtering architectures of these applications, the multipliers cost increase linearly with increasing number of filters of complete designs by using any of the three different filter design approaches. The trend of multiplier cost reduction remains same for our proposed filter design approach as compared to the conventional approach. However for quadrant symmetric approach, the multipliers count is slightly higher for those applications that have non-quadrant symmetric filters due to usage of conventional un-optimized approach for realizing these non-quadrant symmetric filters along with quadrant optimized approach for realizing quadrant symmetric filters. Similarly for serial architectures as shown in Fig 11b, the trend of multipliers cost for conventional and the proposed one remains linear and a single versatile filter design remain sufficient for executing all types of filters sequentially but quadrant symmetric approach needs multiple filters for multiple filter types and able to reduce only few resources due to partial resource sharing among alike filter types.

## Our Proposed method vs FFT based method

The linear filters for image processing as a 2D convolution operation in spatial domain can also be implemented as a point-wise multiplication in frequency domain [10]. However apart from point wise multiplications, the complete filtering operation in frequency domain needs to perform some additional transformation steps. Since the input image and filter is inherently encoded in spatial domain [10, 11] therefore at first the input image and filter need to be transformed from spatial domain to frequency domain by using a Fast Fourier Transform (FFT). Now for filtering, the point wise complex multiplication is performed on these two transformed signals. The output is then need to be re-transformed into spatial domain by using Inverse FFT (IFFT) to acquire the filtered output image. Therefore involves two 2D FFT, one IFFT and point wise multiplications. For a (M × M) input image, the complexity of FFT/IFFT is O(2M^2^log_2_M) [38] and complexity of point wise multiplications is O(M^2^), therefore the total computational cost of complete process will be sum of these operations i.e. O(6M^2^log_2_M+M^2^). This cost is independent of filter size and lower than spatial domain filtering solutions for very large filters.

However they have some inherent limitations such as very high memory consumption [39], rigorous image padding for rounding standard image sizes such as (640 × 480), (1080 × 720), (1080 × 1920) etc. into non-standard square images of the size in power of two [40, 41], requires high bit depth of image pixels for bringing precision during transformation; once from spatial domain to frequency domain and then back to spatial domain which degrades the processing speed [40, 42], etc.

**Fig 12:**
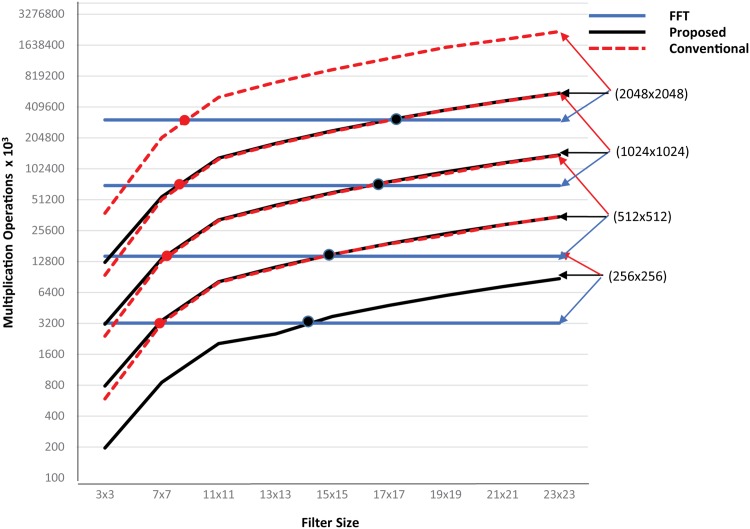
Complexity comparison of proposed method with conventional and FFT based methods.

Even though, due to lower complexity the FFT-based filters are superior to any spatial solution as the filter size increases. The question is which approach is more feasible for which range of filter sizes. Therefore we compared relative effectiveness of both filtering methods (FFT based method in frequency domain and our proposed method in spatial domain) in terms of number of multiplication operations for different image sizes ranging from 256 × 256 to 2048 × 2048 and for filters ranges from 3 × 3 to 23 × 23 size. The Fig 12 shows the feasibility of our method over the FFT method. It indicates that the complexity of our method is lower than FFT for small to mid sized filters of up to 17 × 17 kernel size but for very large filter sizes the FFT-based method is more feasible.

However in practice, many widely used image processing tasks such as noise suppression, image enhancement, edge detection etc. are performed by using well known filters such as Gaussian, Laplacian, Sobel, Prewitt, Frei-Chen, Laplacian of Gaussian etc. All of these filters are mostly available in small kernels. The Sobel, Prewitt, Frei-Chen are typically 3 × 3 filters whereas Gaussian, Laplacian and Laplacian of Gaussian optimally perform in small to mid sizes for most of the image processing tasks [43–45]. Therefore all of these tasks are implemented by using filter sizes within the limit where our proposed method outperforms FFT based method.

It is important to be noticed that our proposed method minimizes the performance gap between frequency and spatial domain filtering. It extend the feasibility of spatial domain over frequency domain for much larger filter sizes. Conventionally spatial domain was feasible in the range of up to 7 × 7 filter size [10] which is now extended up to 17 × 17 filter size by using our proposed filter as shown in Fig 12.

## Conclusion

In this work a low cost structurally optimized yet versatile filter design is presented. A composite symmetric filter structure is proposed that exploits the identities of quadrant and circular *T*-symmetries simultaneously to reduce the multipliers count up to 75% as compared to conventional approach. The framework at the same time empower this composite symmetric structure to incorporate its respective Ψ-symmetry based sub-type and a special asymmetric case. Its capabilities of incorporating these diverse filter types not only offers versatility but also provide a cost effective solution for sequentially implemented image processing applications with diversified filtering requirement. Thus the proposed filter design is highly feasible for efficiently implementing computationally intensive image processing applications, especially in a resource constraint environment with limited computing resources, area and power.
